# Changes in Microbial (Bacteria and Archaea) Plankton Community Structure after Artificial Dispersal in Grazer-Free Microcosms

**DOI:** 10.3390/microorganisms5020031

**Published:** 2017-06-03

**Authors:** Hera Karayanni, Alexandra Meziti, Sofie Spatharis, Savvas Genitsaris, Claude Courties, Konstantinos A. Kormas

**Affiliations:** 1 Department of Biological Applications and Technology, University of Ioannina, 45110 Ioannina, Greece; ameziti@gmail.com; 2 University of Glasgow, BAHCM Institute and School of Life Sciences, Glasgow G12 8QQ, Scotland, UK; Sofie.Spatharis@glasgow.ac.uk; 3 Laboratoire d’Océanologie et Géosciences (LOG), UMR CNRS 8187, Université du Littoral Côte d’Opale (ULCO), 32 av. Foch, 62930 Wimereux, France; savvas.genitsaris@yahoo.gr; 4 Sorbonne Universités, UPMC Univ Paris 06, UMS2348, Laboratoire d’Océanographie Microbienne, Observatoire Océanologique, 66650 Banyuls-sur-mer, France; claude.courties@orange.fr; 5 Department of Ichthyology & Aquatic Environment, University of Thessaly, 383 46 Volos, Greece; kkormas@uth.gr

**Keywords:** microbial assemblage, diversity, community, 16S rRNA, microcosms, pyrosequencing, mixing, biogeography, dispersion

## Abstract

Microbes are considered to have a global distribution due to their high dispersal capabilities. However, our knowledge of the way geographically distant microbial communities assemble after dispersal in a new environment is limited. In this study, we examined whether communities would converge because similar taxa would be selected under the same environmental conditions, or would diverge because of initial community composition, after artificial dispersal. To this aim, a microcosm experiment was performed, in which the temporal changes in the composition and diversity of different prokaryoplankton assemblages from three distant geographic coastal areas (Banyuls-sur-Mer in northwest Mediterranean Sea, Pagasitikos Gulf in northeast Mediterranean and Woods Hole, MA, USA in the northwest Atlantic), were studied. Diversity was investigated using amplicon pyrosequencing of the V1–V3 hypervariable regions of the 16S rRNA. The three assemblages were grown separately in particle free and autoclaved Banyuls-sur-mer seawater at 18 °C in the dark. We found that the variability of prokaryoplankton community diversity (expressed as richness, evenness and dominance) as well as the composition were driven by patterns observed in Bacteria. Regarding community composition, similarities were found between treatments at family level. However, at the OTU level microbial communities from the three different original locations diverge rather than converge during incubation. It is suggested that slight differences in the composition of the initial prokaryoplankton communities, resulted in separate clusters the following days even when growth took place under identical abiotic conditions.

## 1. Introduction

The terms “microbe ubiquity” refers to the concept that microorganisms can overcome environmental and spatial constraints and can be dispersed over large distances [1]. This is related to their high cell abundance and small size that increase their dispersal potential [2]. Furthermore, microbes can be transported—and survive—over long distances in several ways e.g. by aerial movement, migratory birds and ballast water [3]. The above properties, high abundance and dispersion potential, are what lead to the assumption “everything is everywhere, but the environment selects” [4], an issue that still provokes intense discussions [5]. The temporal and spatial turnover of aquatic bacterial populations has been attributed to variations of different biotic and abiotic factors, mostly phytoplankton blooms, grazing, viral infections, parasitic relationships, temperature, sunlight and salinity [6,7,8,9] which may lead to predictable patterns of bacterial population dynamics or short lived blooms of specific taxa [8]. Besides the above mentioned parameters, latitude and geographical distance have been also linked, to the observed variability between and within microbial communities [10], suggesting that microorganisms exhibit biogeographical patterns. This finding contradicts the “everything is everywhere” hypothesis. The importance of environmental conditions on community assembly has also been highlighted in a field study across the Atlantic Meridional Transect, which showed striking similarities in phytoplankton community structure between the same latitude regions, to the north and south of the equatorial divergence [1]. These authors suggested local environmental selection of broadly dispersed species to primarily control phytoplankton community structure. However, more recently, data derived from Tara ocean expedition indicated that factors involving microbial interactions are better predictors of community structure compared to environmental parameters [9].

The way that aquatic microbial communities assemble after dispersal in the new environment is under investigated. A reason for this is the conceptual challenges associated to the study. The main approach followed so far is “transplant experiments” [11,12] in which, water from a particular source (e.g., saline/freshwater, epilimnion/hypolimnion) is incubated in an adjacent area with different environmental conditions. These experiments showed that bacterial community composition is source-dependent but also modulated—to a lower degree—by local conditions. It should be noted that studies on microbial assembly usually involve Bacteria (e.g., [13,14,15]), while mechanisms driving Archaea community composition remain relatively understudied (e.g., [16,17]). In a cross-habitat investigation dealing with both assemblages, it has been shown that Archaea have a distinct assemblage structure to Bacteria, as they followed different relative abundance distributions, which was also reflected in alpha diversity indices [18].

In this study, we investigated how prokaryoplankton (heterotrophic Bacteria and Archaea) from distant geographic areas (≥1712 km) assemble after artificial migration in a new environment. Our question is whether the communities will converge or diverge after incubation under the same environmental conditions, i.e., whether the same ubiquitous taxa are favoured by the prevalence of some specific environmental conditions. We hypothesize that if each ecosystem contains a seedbank of prokaryotes imported by dispersal but not able to thrive in that particular ecosystem (i.e., there is ubiquitous dispersal [2]), then, prokaryoplankton communities will show similarities in their composition after incubation under the same conditions (i.e., eventually the same taxa will be favored in all communities). For this, we performed a growth experiment in which three inocula from the northwest and northeast Mediterranean Sea and the northwest Atlantic Ocean were each mixed with sterile water from the northwest Mediterranean. Microbial plankton community (prokaryoplankton) composition was studied by an OTU (operational taxonomic unit)-based analysis of Bacteria and Archaea assemblages during the lag phase, exponential growth and the stationary phase. Hence, this study investigates not only geographically-distant microbial plankton community composition during growth, but also whether the assembly processes differentiate between Bacteria and Archaea.

## 2. Materials and Methods

### 2.1. Microcosm Preparation

For the growth experiment, nine 20 L autoclaved polycarbonate carboys with spigot were filled with seawater from a coastal site in Banyuls-sur-mer (northwest Mediterranean Sea, FR). To avoid filter clogging due to filtering of a high volume of seawater, sequential filtering through 180, 20 (nylon filters, Millipore, Billerica, MA, USA) and 0.7 (GF/F Whatman filters) μm using a peristaltic pump was performed. Seawater was transferred to the experimental carboys and autoclaved at 121 °C for 15 min. The seawater from Banyuls-sur-mer was then separately inoculated with a natural prokaryoplankton community from the northeast Mediterranean Sea (Pagasitikos Gulf-Greece, coded as P, N 39°13’45’’, E 22°57’12’’), the northwest Mediterranean Sea (Banyuls-sur-mer-France, coded as B, N 42°29’0.3’’, E 3°7’44’’) and the northwest Atlantic Ocean (Woods Hole, MA, USA, coded as W, N 41°31’35.3’’, W 70°40’23.1’’). Samples served as inocula were taken from sea surface and water temperature was <7 °C in Woods Hole while in Banyuls-sur-Mer and in Pagasitikos temperature exceeded 15 °C. Inocula from Pagasitikos and Woods Hole were transferred in 3 × 2 L polycarbonate bottles, at <4 °C in the dark and delivered in 2 days after sampling. Inocula from each sampling site were mixed and filtered through 0.7 μm pore size filters prior to addition and represented 10%, i.e., 2 L, of the final volume (20 L) in each carboy. Microcosms were prepared in triplicate for each inoculum. Banyuls microcosms (i.e., the indigenous community) were considered as “control” throughout the study. Incubation lasted 21 days and took place at 18 °C in the dark. Seawater was gently hand-shaken (overturn and swirled) twice a day during incubation as well as before each sampling. The experiment was conducted from April to May 2011 at the Observatoire Océanologique, Banyuls-sur-mer, France in the frame of the ASSEMBLE project.

### 2.2. Flow Cytometry

For prokaryoplankton cell abundance determination, 5 mL samples were fixed with 2% paraformaldehyde and stored in liquid nitrogen until analysis with flow cytometry. Before counting, samples were thawed at room temperature and nucleic acids were stained with SYBRGreen-I and incubated in the dark for 15 min. Stained bacterial cells were enumerated according to their right-angle light scatter (RALS) and green fluorescence (FL1) collected at 530/30 nm. Counts were performed with a FACSCalibur flow cytometer (Becton Dickinson, San Jose, CA, USA) equipped with an air-cooled 488 nm argon laser. In order to calibrate the flow cytometer, fluorescent beads (1.002 μm; Polysciences Inc., Warrington, PA, USA) were systematically added to each sample. Sampling for prokaryoplankton enumeration was performed at one to two days interval. In total, 11 samples were taken between d0 and the end of the experiment (d21). Bacterial growth rates (μ in d^−1^) were measured considering bacterial abundance (N) at the beginning (t_in_) and the end (t_final_) of the exponential growth in each microcosm [μ= ln(N_final_/N_in_)/(t_final_ − t_in_)]. 

### 2.3. DNA Extraction and Pyrosequencing Analysis

DNA samples were collected from all replicates at the beginning of the experiment (d0), towards the end of the exponential growth phase (d5) and towards the end of the incubation period (d17). Water samples (0.5–1.0 L) were filtered through 0.2 μm pore size polycarbonate isopore filters under low vacuum (≤100 mmHg). Filters were stored immediately at −80°C until further analysis. For DNA extraction, filters were sliced with sterile scalpel and DNA was extracted using the UltraClean Soil DNA isolation kit (MoBio Laboratories, Carlsbad CA, USA) according to the manufacturer's protocol.

Prokaryotic diversity (Bacteria and Archaea) was studied using amplicon pyrosequencing of the V1–V3 hypervariable regions of small-subunit ribosomal RNA genes. For amplicon pyrosequencing, bacterial (27F, 5’-AGRGTTTGATCMTGGCTCAG-3; 519R, 5’-GTNTTACNGCGGCKGCTG-3’) and archaeal (ARC349F, 5’-GYGCASCAGKCGMGAAW-3’; ARC915R, 5’-GTGCTCCCCCGCCAATTCCT-3’) primers were used as previously described [19]. In brief, a one-step 30 cycles PCR was applied using HotStarTaq Plus Master Mix Kit (Qiagen, Valencia, CA). PCR conditions included: 94 °C for 3 min, then followed by 28 cycles of 94 °C for 30 s; 53 °C for 40 s and 72 °C for 1 min; and a final elongation step at 72 °C for 5 min. Following PCR, all amplicon products from different samples were mixed in equal concentrations and purified using Agencourt Ampure beads (Agencourt Bioscience Corporation, MA, USA). Samples were sequenced utilizing Roche 454 FLX titanium instruments and reagents after following manufacturer’s guidelines. Sequencing raw data were processed using MOTHUR v.1.33.0 [20]. The flowgrams of each sample were distinguished according to their tag and were denoised with the PyroNoise software [21]. After removing primer sequences, tag, and key fragments, only sequences with ≥200 bp having homopolymers <8 bp were kept for further analysis. Chimeras were recognised using UCHIME [22] and discarded. The remaining sequences were clustered using a 97% similarity cut-off. Singletons, i.e., sequences that occurred only once in the whole dataset, were also removed from further analysis [23]. Taxonomic affiliation was based on the SILVA 111 SSU RNA database [24]. Sequences from this study have been submitted to the GenBank BioProject (accession number PRJNA305848). Data were normalized to the number of sequences in the smallest group.

### 2.4. Analysis of Prokaryoplankton Community Structure

Prokaryoplankton community structure was expressed through OTU richness, cumulative OTU richness, abundance and dominance expressed by the McNaughton dominance index [25] as the ratio of the dominant species divided by the total number of reads. OTU richness and dominance have been previously found to adequately describe prokaryotic community diversity [18].

General linear models were used to test for the effects of growth phase (factor variable with three levels: day 0, 5 and 17) and site of origin (factor with three levels: Banyuls, Pagasitikos and Woods Hole) and their interaction, on OTU richness, cumulative OTUs and dominance. This analysis was carried out in R v 3.3.2 and the graphs were produced using ggplot. To investigate similarities between our samples within the Archaea and Bacteria assemblages, we used the Bray-Curtis similarity index on OTU presence/absence data and visualised the data with Multidimensional Scaling ordination (MDS). These analyses were carried out with the Primer package v 6.1.16. 

Finally, OTUs were classified as rare (<0.1%), common (0.1–1%) and abundant >1% in relation to their relative abundance in each sample [26]. The Levin’s index (B) was calculated for all OTUs found during the experiment [27]. The B index ranged between 1 and 6 and OTUs were arbitrary classified as generalists when B was higher than 5 and as specialists when B was lower than 2.

## 3. Results

### 3.1. Prokaryoplankton Abundance

**Figure 1:**
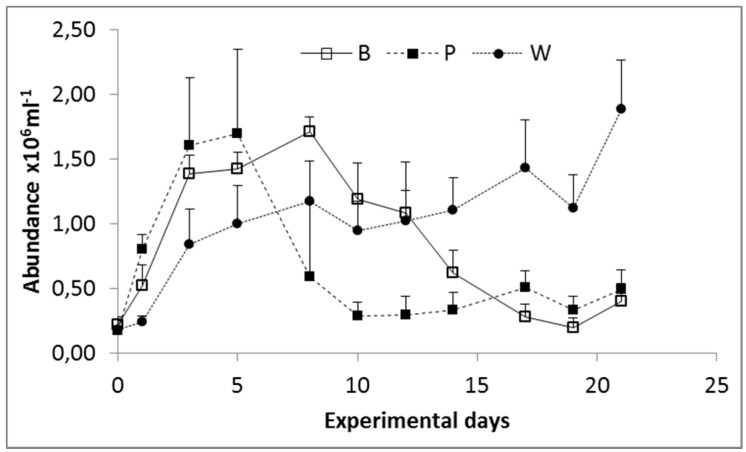
Prokaryoplankton abundance during the experiment. Letters indicate the site of origin of the inoculum. B: Banyuls, P: Pagasitikos, W: Woods Hole.

Prokaryoplankton abundance varied over one order of magnitude (10^4^ to 10^6^ cells ml^−1^) during the course of the experiment (Figure 1). Exponential growth was recorded at the beginning of the experiment in all carboys and lasted between five (Pagasitikos) and eight days (Banyuls and Woods Hole). During the growth phase bacterial abundance reached 1.71 ± 0.11 × 10^6^ cells ml^−1^ in Banyuls, 1.69 ± 0.65 × 10^6^ cells ml^−1^ in Pagasitikos and 1.17 ± 0.35 × 10^6^ cells ml^−1^ in Woods Hole. The highest growth rate was measured in Pagasitikos (0.46 d^−1^). In Banyuls and Woods Hole growth rates were 0.26 d^−1^ and 0.22 d^−1^ respectively.

### 3.2. Prokaryoplankton Community Structure

**Figure 2:**
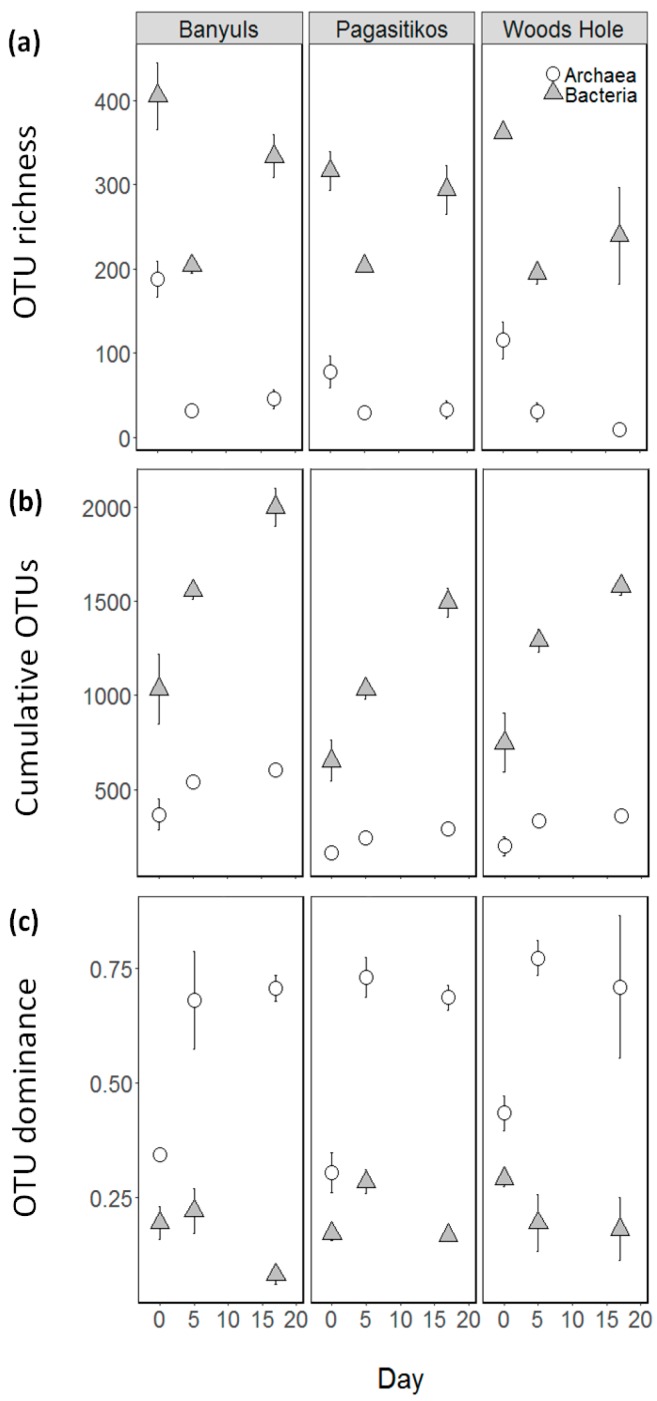
Means with standard errors of OTU (**a**) richness, (**b**) cumulative OTU richness, and (**c**) dominance for Bacteria (grey triangles) and Archaea (white circles) throughout the course of the experiment (day 0, day 5-exponential phase, day 17-stationary phase) in the three different sites of origin (Banyuls, Pagasitikos and Woods Hole).

A total of 4434 unique OTUs (97% sequence similarity) were identified during the study, corresponding to 1150 Archaea and 3284 Bacteria OTUs. Bacteria and Archaea richness presented a significant drop after day 0 and this was consistent regardless of the inoculum site of origin (Figure 2a, Table 1). Interestingly, Bacteria richness presented a partial recovery at the stationary phase, unlike Archaea OTU which remained at similar levels as the exponential phase. The OTU richness turnover across the consecutive growth phases within each site of origin was much greater in Bacteria assemblages as indicated by the steepest curves in cumulative OTU richness (Figure 2b). In Bacteria many new OTUs (~400) were added in each consecutive growth phase whereas for Archaea this number was stabilized (~25) from the exponential stage and onwards.

Bacteria and Archaea dominance were both affected by the growth phase (Figure 2c and Table 1) with Archaea presenting an increasing pattern after day 0, whereas Bacteria a respective decreasing pattern. The increasing Archaea dominance was due to two OTUs (OTU0007 and OTU0003) which dominated across all treatments from day 5 onwards. 

**Figure 3:**
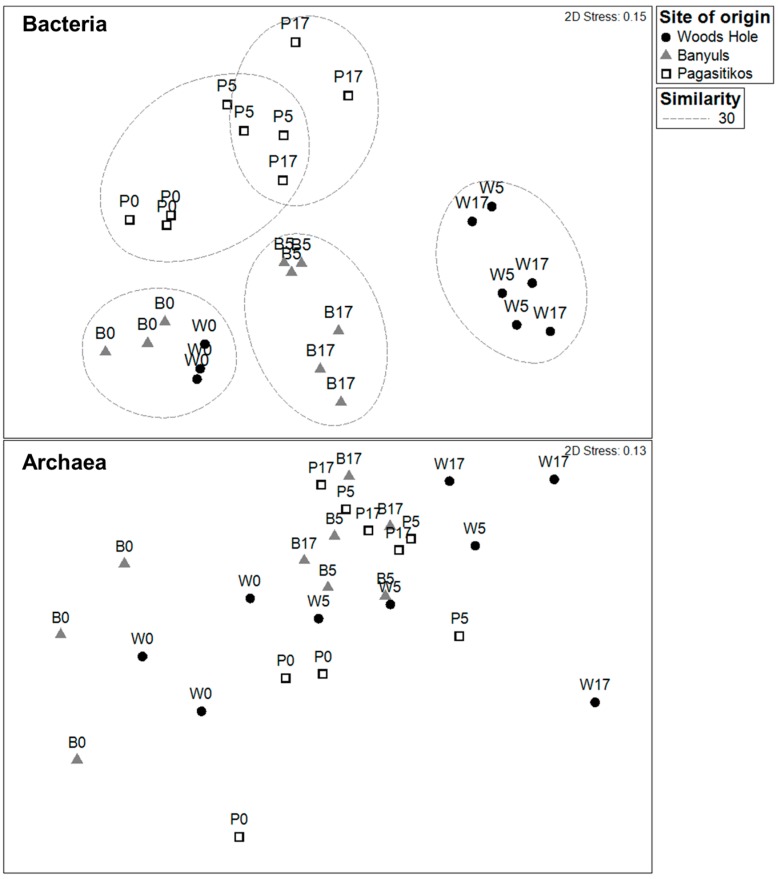
Multidimensional Scaling ordination (MDS) showing the similarity of samples based on the Bray Curtis index on presence-absence Bacteria and Archaea OTU data. Symbols indicate the site that the inoculum originates from (B: Banyuls, P: Pagasitikos, and W: Woods Hole) whereas the labels indicate also the growth phase (day 0, day 5-exponential growth, day 17-stationary phase).

The Bacteria OTU assemblage composition at day 0, was quite similar between Banyuls and Woods Hole with Pagasitikos lying further apart (Figure 3). During the exponential (d5) and the stationary phase (d17) samples from the same site tended to group together and further apart from day 0 which agrees with data from Figure 2b, highlighting the addition of extra OTUs. The Archaea assemblage composition presented a random ordination pattern not related to growth phase or site of origin. The pattern of sample similarities when considering the whole of the prokaryoplankton community (data not shown) was closely following the pattern based on Bacteria similarities (Figure 3). Despite employing the most powerful transformation on our OTU data (i.e., using presence/absence data) we were still able to see a clear separation of bacterial assemblages with respect to the site of the origin indicating that it was the OTU composition and not only the dominant species that changed during the course of the experiment. The Venn diagram (Figure S1) showed also that Banyuls and Woods Hole microcosms shared initially a higher number of bacteria and archaea OTUs compared to Pagasitikos. It was also demonstrated that the number of OTUs that overlapped among the three microcosms decreased while the fraction of unique OTUs in each treatment increased during the course of the experiment (Figure S1).

**Figure 4:**
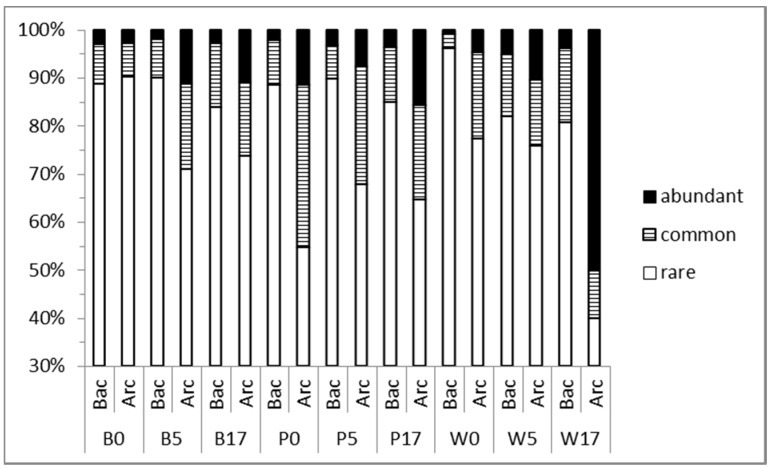
Relative abundance of abundant (>1%), common (≥0.1% to <1%) and rare (<0.1%) Bacterial (Bac) and Brchaeal (Arc) OTUs in the three different sites at day 0, day 5-exponential growth, day 17-stationary phase. B: Banyuls, P: Pagasitikos, W: Woods Hole.

The number of abundant bacterial OTUs (relative abundance >1%) during the course of the experiment was 7–14, 11–17 and 14–15 for Banyuls, Pagasitikos and Woods Hole respectively. These OTUs represented ≤1% of the total number of OTUs (Figure 4) but 73–91% of total reads in different treatments and sampling days (except in Woods Hole microcosm at d0, 55%). Table S1 indicates the changes of the relative abundance of the most abundant OTUs found at the beginning of the experiment, the growth phase and the end of the experiment in different microcosms. In Banyuls and Pagasitikos microcosms abundant Bacterial OTUs at d5 originated mainly from the pool of the initially abundant and/or common OTUs (Figure 4). On the contrary in Woods Hole microcosms initially rare OTUs increased in abundance and dominated the Bacterial assemblage. At day 17 the majority of the abundant Bacterial OTUs found in different microcosms were rare at the beginning of the experiment. Dominant Archaeal OTUs at d5 and d17 were ubiquitous and abundant at d0, except in Woods Hole at d17 where an initially undetected and then rare OTU became abundant and one of the dominant OTUs. The number of generalists found throughout the study was very low (8) and only two of them (OTU1 and OTU2) were among the abundant OTUs at least in one microcosm. Specialists represented 95% of the total number of OTUs found throughout the study. 

### 3.3. Taxonomic Diversity at the Family Level 

**Figure 5:**
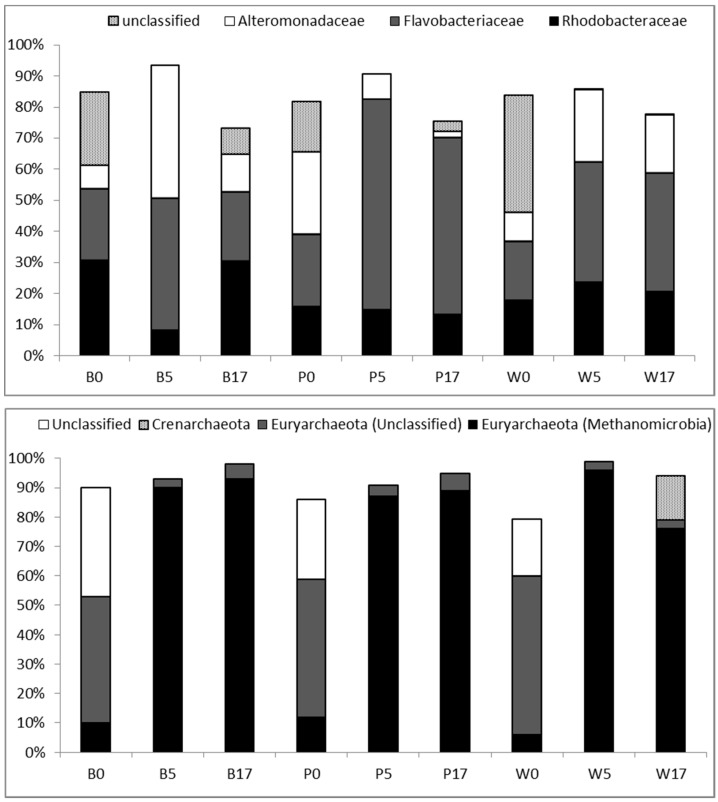
Relative abundance of dominant Bacterial families (cumulative 70%) and Archaeal phyla (cumulative 80%) in the three different sites at day 0, day 5-exponential growth, day 17-stationary phase. B: Banyuls, P: Pagasitikos, W: Woods Hole.

OTUs (cumulative abundance 70%) belonged mainly to three families *Rhodobacteraceae, Flavobacteriaceae* and *Alteromonadaceae* (Figure 5, Table S1). Overall, these families contributed >60% to total relative abundance except in Woods Hole at d0 (46%). Relatively few (<6%) “abundant” OTUs were grouped under *Piscirickettsiaceae, Chitinophagaceae, Pseudomonadaceae* and *Oceanospirillaceae*. The contribution of unclassified OTUs ranged between 16 and 38% in different treatments at the beginning of the experiment and then dropped to <8%. Closest relatives of dominant OTUs have been mostly detected in surface marine waters and coastal systems e.g., [28,29]. The archaeal assemblage was dominated by Euryarchaeota. The number of archaeal OTUs that reached more than 1% of total reads ranged between 4 and 13 in different microcosms and growth phases (experimental days). Dominant OTUs clustered in Methanomicrobia, in Marine Group II or were unclassified. These OTUs represented always >82% of total sequences’ abundance. Closest relatives of dominant Methanomicrobia and MGII related OTUs had been detected in marine sediments [30] and coastal seawater [16] respectively.

## 4. Discussion

In this study, we investigated the temporal changes of the prokaryoplankton community (Bacteria and Archaea) in artificially mixed microcosms from the day of inoculation (d0) to the stationary phase (d17). The initial communities originated from three distant geographic sites and were grown separately under the same growth medium and incubation conditions. Our results showed that the prokaryotic communities from Woods Hole and Pagasitikos Gulf could grow efficiently and with similar or even higher growth rates (0.22 and 0.46 d^−1^, respectively) compared to the “local” (Banyuls) community (0.26 d^−1^). High growth rates in Pagasitikos indicate probably the presence of limiting factors for the respective bacterial assemblage at in situ conditions. In fact, the inner part of Pagasitikos gulf is considered to be nitrogen limited [31]. Nitrogen limitation in the Bay of Banyuls-sur-mer appears during summer whereas N:P ratio increases in winter-early spring [32]. We should also mention that changes in bacterial abundance, growth as well as community composition discussed below may be related to the confinement (e.g., [33,34]). However, we tried to overcome this limitation by using the largest possible volume incubation vessels (20 L)—vessel size was limited by e.g., water volume to be sterilized—since it has been shown that bottle-effect increases for volumes <1 L [34].

Considering the high variability in bacterial community composition between coastal ecosystems [10], we expected the initial samples from the three treatments to be very different. However, all samples from day 0, and in particular Banyuls and Woods Hole, showed high similarity. Samples from the exponential growth (d5) and the stationary phase (d17) were mostly grouped together and separately from the day 0 samples, as expected after the selection that takes place in the beginning of the growth phase in batch cultures, also probably related to the confinement [33]. Moreover, differences in the dynamics of ubiquitous phylotypes (e.g., OTU1) and the selection for different phylotypes in each treatment (e.g., OTU1 in Banyuls, OTU6 in Pagasitikos, OTU9 in Woods Hole) (Table S1) were evidenced during the experiment. Therefore, we could suggest that slight differences in the composition of the initial bacterial assemblages, resulted in separate clusters the following days even upon growth under identical conditions. 

More than 50% of the common and abundant bacterial OTUs that were found at the end of the experiment (d17) in each microcosm, originated from the pool of the initially rare OTUs. Rare taxa presented high degree of ubiquity, however experimental conditions did not select for the same rare OTUs in the different treatments. These findings highlight their role as active players in bacteria assemblage dynamics [35,36] and suggest that the history and distribution of taxa in the inoculum regulate bacterial community composition [37]. No clustering of samples with respect to growth phase or site of origin was observed based on Archaea OTU composition highlighting different assembly processes to Bacteria.

Both Bacteria and Archaea richness presented a drop at the growth phase indicating a takeover of the microcosms by few abundant OTUs. Partial recover of Bacterial diversity was observed at the end of the experiment. Since grazing pressure was absent in the experimental set-up, this may suggest a virus induced Bacterial mortality after exponential growth, following the “killing the winner” hypothesis , with direct effects on community composition and diversity [38]. Furthermore, this diverse community suggests a resource partitioning between Bacteria or the co-occurrence of OTUs with complementary functions [39] or cross-feeding [40,41]. On the contrary, high dominance in Archaea, at the end of the experiment, indicates resource limitation and significant competitive interactions within this assemblage or with the Bacteria leading to strong selection by specific Archaea species. The two groups share the same dissolved organic carbon pool but Bacteria are probably able to use first the most labile substances. A previous study [42] has shown that a single bacterial strain is able to remove completely the labile carbon in coastal waters and based on this finding it was suggested that diverse organic compounds available for decomposition in marine environments shape bacterioplankton diversity [43]. Overall, Archaea presented higher dominance compared to Bacteria, which is consistent with previous findings across different aquatic ecosystems [18].

The most important classes contributing to the bacterial communities during the course of the experiment were *Flavobacteria, Gammaproteobacteria (Alteromonadaceae) and Alphaproteobacteria (Rhodobacteraceae)*, which are the predominant groups in marine coastal systems [44]. Moreover, *Gammaproteobacteria* have been previously reported as a qualitatively important group, growing rapidly in mesocosm experiments when nutrients become available [14,45]. Based on these findings, we would expect *Alteromonadaceae* to proliferate and dominate during growth. However, although *Alteromonadaceae* increased after inoculation (except in Pagasitikos), they were either equally represented (Banyuls) or outcompeted by *Flavobacteriaceae* (Pagasitikos and Woods Hole). Bacteria of the *Cytophaga-Flavobacteria* group are considered as slow growers limited probably by particulate organic carbon concentration in batch cultures experiments [46]. Therefore, the rise and the predominance of *Flavobacteriaceae* in the different treatments could be regarded as an indication for increased concentration of high molecular weight dissolved organic carbon, particulate carbon or organic sources of nutrients in the growth medium [46,47,48]. So it seems that similarities between the different treatments may occur at the higher taxonomic, family level. Moreover, these findings reinforce the hypothesis that besides confinement and grazing, competition with *Bacteroidetes* may explain the dynamics of *Alteromonadaceae* [45]. In contrast to the above observation, the contribution of Rhodobacteraceae differed between the different treatments. *Rhodobacteraceae* were found to proliferate and dominate only in Banyuls, at the beginning (d0) and the end (d17) of the experiment. A shift toward *Rhodobacteraceae* has been associated to high chlorophyll and bacterial production levels, low salinity and the presence of recalciltrant hydrocarbons [49,50,51]. Their predominance at the beginning of the experiment may be related to one or more of the above parameters, however considering that growth medium and environmental conditions were identical for the three different treatments it is unclear what caused their increase at d17. We could probably suggest that *Rhodobacteraceae* are benefited by biotic interactions and in particular by the competition between *Flavobacteriaceae* and *Alteromonadaceae* during growth. Overall, at higher taxonomic level, we found differences between treatments at day 0, and these differences affected the composition of the bacterial community at the end of the experiment. Moreover, it is noteworthy, that similarities were observed between the two ‘allochtonous’ communities (Pagasitikos and Woods Hole) compared to the “indigenous” one (Banyuls), on what concerns the bacterial taxonomic composition at the family level.

Archaea were present and dominated by Euryarchaeota during our experiment, and this is consistent with previous field studies in coastal waters e.g. [52]. As mentioned earlier Archaea dominance increased during growth (d5). Surprisingly, dominant OTUs were affiliated to the methanogens *Methanosarcinales*. We could claim that in our microcosms Archaea were able to proliferate in a confined space and in particular in the anoxic microhabitats associated to fine particles suspended in the water [53]. Actually, it has been shown previously that water column particles host a diverse community of Archaea and that Crenarchaeota cells in particular are often associated with particles [54]. Therefore, our findings seem to support previous field data that methanogenesis may occur in well oxygenated waters [55]. It is also interesting to note the proliferation of an initially rare (<0.1% relative abundance) and ubiquitous (detected in all treatments) Crenarchaeota affiliated OTU in the Woods Hole microcosms at the end of the experiment (d17). This finding indicates that not only Bacteria but also Archaea can switch from rare to common/abundant following changes in environmental conditions [17] or as a consequence of biotic interactions. 

Concluding, our study showed that clearly distinct communities develop not only when the inoculums are distinctly different [37], but even when slight differences occur between them. Prokaryoplankton communities were differently assembled at the lower taxonomic level (OTU) and did not seem to converge although ubiquitous rare taxa were able to proliferate during the study. Thus, the assembly of the prokaryoplankton community is driven by the inoculum. Furthermore, we could suggest that Bacteria and Archaea are differently assembled and that competitive interactions between/within the two assemblages shape their composition and diversity. It has been suggested that diversity enhances community productivity in new environments and the robustness of community function to environmental perturbation [56]. Our results suggest the presence of a large pool of Bacteria with niche differences and probably with distinctive roles in coastal ecosystems functioning compared to Archaea.

## Figures and Tables

**Table 1:** The effect of the site of origin of the inoculum (Banyuls, Pagasitikos, Woods Hole) and day of the experiment (Day 0, day 5-exponential phase, and day 17-stationary phase) and their interaction, on different aspects of prokaryoplankton community structure (richness, cumulative OTUs and dominance). Single asterisks denote that the null hypothesis (no effect) is rejected at the 0.05 level, double asterisks at the 0.01 level and triple asterisks at the 0.001 level based on the respective F-ratio value.

	F-Ratio		
	Site	Day	Interaction of Site with Day
Richness—all	6.89 *	48.93 **	3.40 *
Richness—Archaea	8.02 **	48.95 **	5.01 **
Richness—Bacteria	2.68	24.42 **	1.34
Dominance—Archaea	1.696	25.73 **	0.27
Dominance—Bacteria	0.83	4.56 **	2.12
Cumulative OTUs—all	4.66 *	16.12 ***	0.08
Cumulative OTUs—Archaea	35.76 ***	22.68 ***	0.67
Cumulative OTUs—Bacteria	11.99 ***	74.08 ***	0.21

## Supplementary Materials

The following are available online at www.mdpi.com/2076-2607/5/2/31/s1, Figure S1: Venn Diagram at distance 0.03, illustrating the number of unique and shared bacterial and archaeal (in parenthesis) OTUs (97% sequence similarity) among Banyuls (B), Woods Hole (W) and Pagasitikos (P) microcosms at d0 (top), d5 (middle) and d17 (bottom); Table S1. Bacteria (cumulative >70%) and Archaea (cumulative >80%) OTUs and families/phyla relative abundance. Bold: dominant OTUs.
