# Supplementary material for: Changes in Microbial (Bacteria and Archaea) Plankton Community Structure after Artificial Dispersal in Grazer-Free Microcosms
Source: Microorganisms. 2017 Jun 3;5(2):31. doi: 10.3390/microorganisms5020031 (PMC5488102; doi:10.3390/microorganisms5020031)
Supplement: Supplementary file 1 [file microorganisms-05-00031-s001.zip › FigureS1.docx]

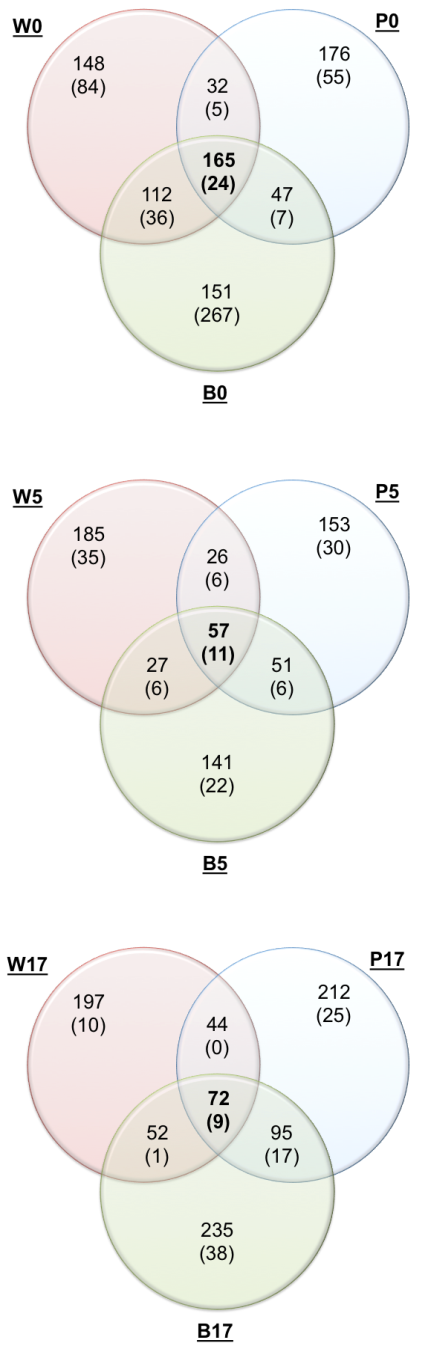


**Figure S1.** Venn Diagram at distance 0.03, illustrating the number of unique and shared bacterial and archaeal (in parenthesis) OTUs (97% sequence similarity) among Banyuls (B), Woods Hole (W) and Pagasitikos (P) microcosms at d0 (top), d5 (middle) and d17 (bottom).
